# Corticospinal vs Rubrospinal Revisited: An Evolutionary Perspective for Sensorimotor Integration

**DOI:** 10.3389/fnins.2021.686481

**Published:** 2021-06-11

**Authors:** Rafael Olivares-Moreno, Paola Rodriguez-Moreno, Veronica Lopez-Virgen, Martín Macías, Moisés Altamira-Camacho, Gerardo Rojas-Piloni

**Affiliations:** Instituto de Neurobiología, Universidad Nacional Autónoma de México, Querétaro, Mexico

**Keywords:** motor control, motor cortex, red nucleus, spinal cord, motor recovery

## Abstract

The knowledge about how different subsystems participate and interplay in sensorimotor control is fundamental to understand motor deficits associated with CNS injury and movement recovery. The role of corticospinal (CS) and rubrospinal (RS) projections in motor control has been extensively studied and compared, and it is clear that both systems are important for skilled movement. However, during phylogeny, the emerging cerebral cortex took a higher hierarchical role controlling rubro-cerebellar circuits. Here, we present anatomical, neurophysiological, and behavioral evidence suggesting that both systems modulate complex segmental neuronal networks in a parallel way, which is important for sensorimotor integration at spinal cord level. We also highlight that, although specializations exist, both systems could be complementary and potentially subserve motor recovery associated with CNS damage.

## Introduction

A fundamental goal of neuroscience is to understand how the brain regulates movement. Motor control in mammals involves multiple descending pathways that form systems which regulate different aspects of movement (Lemon, 2008).

In the last 150 years, there has been a dispute about the functions of corticospinal (CS) and rubrospinal (RS) tracts. Both are descending motor pathways and have remarkably similar functional properties. It has been proposed that each system is primarily active in different movement contexts. Hence, the CS tract is most involved when a new movement is being learned, while the RS tract is preferentially active when automated movements are being executed (Kennedy, 1990). However, what hierarchically superior structure decides which system should be in use? Do these two systems represent separate ways to perform skilled movements depending on the movement needs throughout evolution? Moreover, is the information carried by these systems redundant or complementary? And how could the RS tract improve skilled movements when the CS tract is injured or absent? Furthermore, which spinal cord neuronal circuits and neurons are modulated by rubral and cortical descending projections?

In this review, some of these questions will be addressed to understand in an integrative way the functional hierarchy of both systems in the context of sensorimotor control, which is particularly important, since RS systems have been considered vestigial in humans.

## Rubrospinal System in Primitive Vertebrates

Throughout the evolution of vertebrates, different locomotor patterns such as swimming, crawling, walking, running, jumping, brachiation, flying, and burrowing were developed. Each of these diverse vertebrate locomotor modes is derived from the fundamental swimming pattern, present in most aquatic vertebrates (Lindsey, 1978; McClellan, 1988; Grillner, 2018).

From lamprey to skate fish and primates, the locomotor system is organized in a similar way, with a midbrain locomotor command region that activates spinal circuits responsible for generating the motor pattern. These descending pathways to the spinal cord represent the instruments by which the central nervous system steers diverse locomotor modes. Direct telencephalo-spinal pathways, such as the mammalian CS tract, are absent in primitive vertebrates. However, in origin, course, and site of termination, descending brainstem pathways show remarkable similarities in amphibians, reptiles, birds, and mammals, suggesting a phylogenetic constancy of descending input from the brainstem to the spinal cord in vertebrates. This implies that many aspects of the basic design for locomotion in the vertebrate nervous system had already evolved at the dawn of vertebrate evolution (Ten Donkelaar, 1982; Grillner, 2018).

In ancient vertebrates, the descending pathways are formed by the reticulospinal tract. This system constitutes the most primitive mechanism involved in motor control in all vertebrates from cyclostomes to mammals (Williams, 1986; McClellan, 1988; Dimitrijevic, 1998). With the appearance of extremities, the development of a suitable neural control system for the steering of limb movements became apparent. It seems likely that the RS tract plays an important role in this mechanism. Interestingly, as reviewed later, a red nucleus (RN) and its efferent pathway, i.e., the RS tract, are present in primitive vertebrates (Ten Donkelaar, 1982).

**FIGURE 1:**
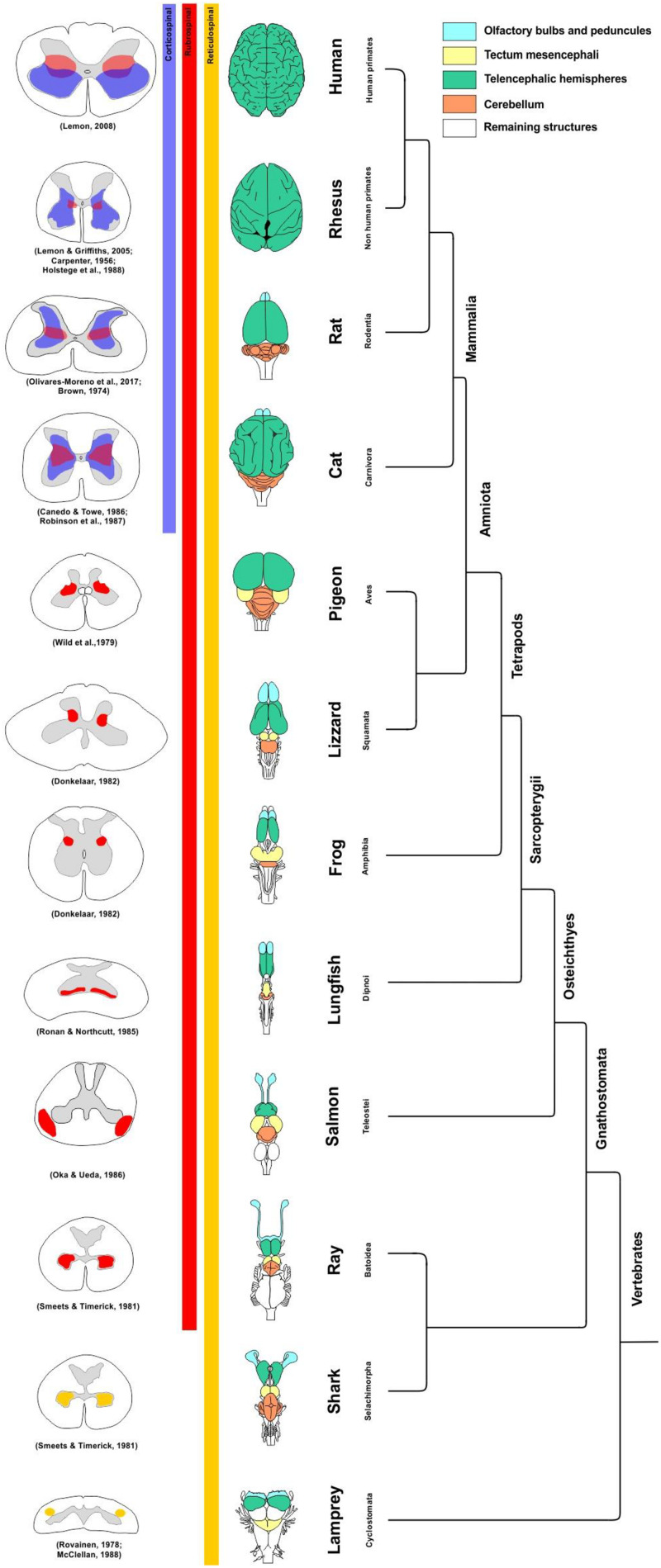
Phylogenetic relationship among the main groups of vertebrates. The drawings show a dorsal view of the brain of representative species of each group, as well as a transverse section of the spinal cord. Ancient vertebrates without RS and CS projections received only descending reticulo-spinal projections (yellow). The shadows in the spinal cord gray matter indicate the zones where rubrospinal (red) and corticospinal (blue) fibers terminate in each group. The data supporting the RS and CS terminals in the spinal cord are indicated (Carpenter, 1956; Brown, 1974; Rovainen, 1978; Wild et al., 1979; Smeets and Timerick, 1981; Ten Donkelaar, 1982; Ronan and Northcutt, 1985; Oka et al., 1986; Robinson et al., 1987; Holstege et al., 1988; McClellan, 1988; Lemon, 2008; Olivares-Moreno et al., 2017).

The presence of the RN throughout vertebrate evolution has been reviewed by ten Donkelaar (1988) and more recently by Basile et al. (2021). Horseradish peroxidase (HRP) studies have revealed that the RN pathway is present in most vertebrate groups. However, in agnathans, this structure has not been identified (Pombal and Megías, 2011). The presence of RN was possible to determine in some species of *chondrichthyans* (cartilaginous fishes), such as rays (*Raja clavata*), with the injection of HRP in the 20th segment of the spinal cord in both the ventral and the dorsal horn (Smeets and Timerick, 1981). However, the pathways and precise ending sites remain unknown (Figure 1; Smeets et al., 1983).

In *Osteichthyes* (actinopterygians and sarcopterygians), the closest relatives of chondrichthyans, the RN can also be seen. In actinopterygians (e.g., Salmon *Oncorhynchus nerka*), two candidates of RN can be observed and one of them could be homologous to other vertebrates (Oka et al., 1986; Nakayama et al., 2016). Thus, the RN sends axons to the ventrolateral region of the spinal cord (Figure 1). However, its role in fine motor control remains to be studied (Yamamoto et al., 2017).

In dipnoans (*sarcopterygians*), a type of lungfish and the closest relatives of tetrapods, the RN has been detected with HRP implants in cervical segments of the spinal cord (Ronan and Northcutt, 1985). Thus, it seems that the presence of RN is a first trial of evolution for the control of appendages for locomotion in water, at least in rays and lungfishes. On the other hand, in other vertebrates with undulating movements, such as lampreys (Pombal and Megías, 2011) and sharks (Smeets and Timerick, 1981), the RN has not been found, nor has its function in teleosts (Yamamoto et al., 2017). Likewise, this nucleus has not been found in some limbless tetrapods, such as caecilian (*Ichthyophis kohtaoensis*) (Naujoks-Manteuffel et al., 1988) and snakes (*Python reticulatus*) (Ten Donkelaar, 1976). This suggests an important role of RN in limb movement control (Ten Donkelaar, 1982). Nevertheless, Sánchez-Camacho et al. (2001) found RN in all three groups of amphibians, including caecilians, urodeles, and anurans. In anurans (*Xenopus laevis*), the RS tract ends in the lateral portion of the spinal cord, being very similar to that of the lizard (*Tupinambis nigropunctatus*) that ends in laminae V and VI (Figure 1; Ten Donkelaar, 1982). Likewise, in birds (*Columba livia*), the RN sends projections to the lateral portion of the spinal cord, in laminae V and VI, both at the cervical and at the lumbar level (Figure 1; Wild et al., 1979). In marsupial mammals, such as opossum (*Didelphis virginiana*), it appears that the lateral arrangement of the RN endings around laminae V and VI is preserved throughout the entire spinal cord (Martin and Dom, 1970).

The neuroanatomical studies performed in primitive mammalian and non-mammalian vertebrates suggest a common ancestor with limb locomotion skills related to similar projections of the RN to the spinal cord, supporting the idea that the genetic machinery was acquired before the development of real limbs (Jung et al., 2018). Nevertheless, studies are required to investigate the functional relevance of this projection in these groups of vertebrates and review the case of actinopterygians and the loss of this projection in some limbless vertebrates.

## Rubrospinal System in Mammals (Eutheria)

The RN has been extensively studied in rodents, cats, and monkeys. In rodents and cats, this system consists of two main regions without a clear anatomical division: The magnocellular region comprises thick Nissl bodies, more caudal, with giant (>40 μm) and large (26–40 μm) cells. In contrast, the parvocellular region contains thin and less conspicuous Nissl bodies, located rostrally, with small (<20 μm) and medium (20–25 μm) cells (Pompeiano and Brodal, 1957; Reid et al., 1975; Strominger et al., 1987; Liang et al., 2012). The RN contains a third population of cells, called interneurons, which are the smallest and are distributed throughout the nucleus (Reid et al., 1975; Strominger et al., 1987). The magnocellular region receives projections from the nucleus interpositus of the contralateral cerebellum with a specific topographical organization (Jansen and Jansen, 1955; Angaut and Bowsher, 1965; Courville, 1966; Daniel et al., 1988) making monosynaptic contacts with or near the soma (Tsukahara and Kosaka, 1968). The parvocellular region projects to the ipsilateral lower olive, which in turn projects to the cerebellum (Massion, 1967; Edwards, 1972; Walberg and Nordby, 1981; Kennedy, 1990). The parvocellular zone receives afferents from the dentate nucleus of the cerebellum (Angaut and Bowsher, 1965; Courville, 1966) and is directly innervated by the cortex through the corticorubral pathway (making monosynaptic contact with the distal dendrites of the RN neurons) and by collaterals of the pyramidal tract and CS neurons (Tsukahara et al., 1967, 1968; Brown, 1974; Gwyn and Flumerfelt, 1974; Canedo and Towe, 1986).

The ventromedial part of the magnocellular region of the RN is the main origin of the RS tract (Massion, 1967; Liang et al., 2012). Due to the lack of a clear anatomical division between magnocellular and parvocellular areas of the RN, some authors have shown that the parvocellular region also contributes to the RS tract in rodents and cats (Huisman et al., 1981, 1983; Shieh et al., 1983). A somatotopic organization has been described between the projections of the magnocellular region and its projections to the spinal cord, with the medial regions projecting to the cervical area and the ventromedial to the lumbar area of the spinal cord (Murray and Gurule, 1979; Shieh et al., 1983). Furthermore, RS projections show a specific distribution to the spinal cord, the parvocellular region projecting to upper cervical regions (controlling proximal muscles) and the magnocellular region to lower cervical regions (controlling distal muscles) (Horn et al., 2002; Pong et al., 2002).

The RS tract decussates in the ventral midbrain tegmentum, descending contralaterally through the dorsolateral funiculus. The axons end at the lateral part of the intermediate zone (laminae V to VII) at all levels of the spinal cord (Strominger et al., 1987; Figure 1), where it makes connections mainly with dendritic branches of premotor interneurons (Kostyuk and Skibo, 1975). However, in rats, RS projections to ventral layers of the spinal cord were also described, making direct contact with motor neurons and participating in the control of intermediate (forearm) and distal muscles (leg, flexor, and abductor digits) (Küchler et al., 2002).

### RN in Primates

The RN in primates is a very extensive ovoid-like structure near the cerebral aqueduct in the caudal portion of the midbrain, which is located between the cerebral aqueduct of Sylvius and ventral to the *substantia nigra*, similar to the location of the RN in man (Cacciola et al., 2019). The RN has an average volume of 10.9 mm^3^ in the rhesus monkey (Carpenter, 1956), about 8.38 mm^3^ in *Macaca mulatta* (King et al., 1971), around 5.99 mm^3^ in *Macacus fuscatus* and 6.28 mm^3^ in *Macacus cyclopis* (Fukuyama, 1940), and 8 mm^3^ in the macaque (*Macaca fascicularis*) (Burman et al., 2000).

The literature shows that the RN is particularly important to muscle control of the limbs in primates (Larsen and Yumiya, 1980; Gibson et al., 1985; Kennedy et al., 1986; Mewes and Cheney, 1994; Miller and Houk, 1995); however, there are clear anatomical differences between quadrupeds and slightly upright primates with bipedal locomotion. An example of this can be observed comparing the anatomical differences between baboons and gibbons. In baboons, the number of RS cells controlling the hindlimb is slightly larger than the population controlling the forelimb (Padel et al., 1981). Moreover, the RN volume in baboons is around 2.93 mm^3^ compared with 1.54 mm^3^ in gibbons (Padel et al., 1981). The number of cells degenerating after the thoracic spinal cord section in gibbons is about two-fifths the amount in baboons with a similar level of section, suggesting that there is further impoverishment of the RN cell populations controlling the hindlimb in an anthropoid with a mainly erect posture and bipedal locomotion like the gibbon (Padel et al., 1981). Thus, together with the development of the bipedal stance and with a greater use of arm and hand in movements in the surrounding space, it seems that forelimb movements of primates are controlled by more recent central structures, such as the pyramidal tract and the neocerebellar-cortical loop.

The RN in primates, humans, and non-humans is also structurally and functionally divided into two main regions: magnocellular and parvocellular (Hatschek, 1907). In cats or rats, this division is not so evident. The parvocellular region in human and non-human primates is more developed, and the magnocellular region has been considered vestigial (Hicks and Onodera, 2012). The RN in primates also receives substantial projections from the motor, premotor, and frontal regions of the cerebral cortex and cerebellum, and projects to cerebellum, inferior olive, and spinal cord (Massion, 1967).

The parvocellular region of the RN is prominent in primates compared with other species (Massion, 1988). Through its topographic organization, it sends projections to the dorsal lamina of the inferior olivary nucleus (Flumerfelt et al., 1973); thus, the lateral part of the parvocellular RN sends projections to the medial part of the dorsal lamina, and the dorsal area sends projections to medial parts of the ventral lamina (Courville and Otabe, 1974; Strominger et al., 1979). In turn, these areas of the olivary nucleus project to the lobulus simplex and crus I and II in the cerebellum (Carpenter, 1956). From the cerebellum, two areas project to the RN in the monkey: the dentate nucleus and the nucleus interpositus anterior. The first projects to the parvocellular RN and the second to the magnocellular RN (Flumerfelt et al., 1973).

The RN in the monkey receives ipsilateral projection from cortical neurons in prefrontal and parietal areas (M1, supplementary motor areas) that are distributed in layer V (Humphrey and Rietz, 1976; Catman-Berrevoets et al., 1979). The most abundant corticorubral projection ends at the parvocellular RN, and only a few neurons project to the magnocellular zone. These corticorubral fibers also show a somatotopic distribution in the RN; the represented upper parts of the body in the parietal cortex end in the magnocellular RN, and the caudal region of the body is represented in the parvocellular RN (Humphrey et al., 1984). It is clear that the RN has a somatomotor organization where the most dorsal region controls forelimb movements and the most ventral part controls hindlimb movements (Burman et al., 2000).

The RS tract in primates originates from the magnocellular region of the RN projecting *via* the lateral funiculus to the intermediate zone of the spinal cord, mainly between C8 and T1 segments (Kuypers et al., 1962). The magnocellular area of the *M. mulatta* contains mostly giant neurons. The size of the cell bodies is between 50 and 90 μm, and the cytoplasm contains abundant chromatin (King et al., 1971). These neurons are multipolar stellate cells and form part of the RS system (Burman et al., 2000). Additionally, magnocellular RN has medium-sized neurons (30–40 μm) (King et al., 1971). However, the predominant neurons in the parvocellular RN are oval and medium-sized (20–30 μm); they contain a granular cytoplasm and have a triangular or fusiform shape. The RN also comprises small neurons (10–15 μm), which contain a small amount of cytoplasm and are equally distributed in both parts of the RN (Miller and Strominger, 1973).

### Red Nucleus in Humans

The RN in humans is a structure in the middle of the midbrain, below the aqueduct of Sylvius, and next to the *substantia nigra* (Milardi et al., 2016). It is known that the RN in humans receives afferents from the dentate nucleus of cerebellum and the cerebral cortex (Habas and Cabanis, 2007); however, new techniques like diffusion tensor imaging (DTI) provide anatomical information about several regions of the cerebral cortex that connect to the RN, including the prefrontal, sensorimotor, premotor lateral and medial prefrontal, and cingulate cortices. Also, the RN has anatomical connections to the thalamus, paracentral lobule, precentral gyrus, and superior frontal gyrus, and is less connected to the caudal middle frontal gyrus, inferior and superior parietal lobules, and middle temporal gyrus (Hirata et al., 2015; Milardi et al., 2016).

The magnocellular part of the human RN is located in the rostral part and is usually considered vestigial in humans (Basile et al., 2021). This area possesses large neurons (Onodera and Hicks, 2009) that give rise to the RS tract, which crosses the midline by ventral tegmental decussation (Yang et al., 2011) and culminates at the contralateral side of the upper cervical segments (Nathan and Smith, 1982). The parvocellular RN is more developed in humans than the magnocellular subdivision; it is located rostrally, contains medium and large neurons (Onodera and Hicks, 2009), and projects to the ipsilateral olive in the central tegmental tract.

The human RN plays a critical role in motor control and is involved in the regulation of muscle tension, motor responses, motor learning, and sensory discrimination (Liu et al., 2000). Recently, it has been associated with cognitive functions related to salience detection, executive control (Hirata et al., 2015; Cacciola et al., 2019), and emotion processing (Zhang et al., 2015).

### Functions of the RN

The function of the magnocellular region of the RN is well characterized in quadruped animals. It has been shown that in both, rats and cats, there are changes in the activity of RS neurons related to the beginning of the execution of voluntary movements of the anterior and posterior limbs (Ghez and Kubota, 1977; Burton and Onoda, 1978; Amalric et al., 1983; Batson and Amassian, 1986; Schmied et al., 1988; Jarratt and Hyland, 1999). The magnocellular region has been implicated in behaviors such as scratching, locomotion, and learned or automated motor behaviors (Kennedy, 1990; Gruber and Gould, 2010). On the other hand, the functional role of the parvocellular region originating rubro-olivary tracts is not yet clearly known. It has been described that cortico-rubro-olivary system is associated with different functions like movement learning and antinociception (Kennedy, 1990; Gruber and Gould, 2010), and proposed that act as a switch from automated movements (regulated by magnocellular RN and RS tracts) to movement learning, mediated by CS tract and olivocerebellar system (for review, see Basile et al., 2021).

Some authors have attributed differential control by the RN to the different muscles of the limb, suggesting that it has a greater influence on distal than on proximal muscles (Ghez and Kubota, 1977; Burton and Onoda, 1978; Amalric et al., 1983; Küchler et al., 2002). However, other authors have shown that it is not possible to see these differences with respect to the control of muscle groups, and the difference in activity is given by the type of movement being performed (Whishaw et al., 1990, 1998; Muir and Whishaw, 2000). In this way, it has been observed in the cat that the RN is related to the maintenance and correction of body posture and voluntary modification of gait (Rho et al., 1999; Lavoie and Drew, 2002; Zelenin et al., 2010), as well as with the rhythmic movement of the jaw (Satoh et al., 2007). On the other hand, activity in the RN in cats (Orlovsky, 1972; Arshavsky et al., 1988) and rats (Muir and Whishaw, 2000) has been associated with different phases of locomotion, and a deficit in this movement has been described when there is damage to this structure. Additionally, in rodents, the RN is involved in the control of skillful movements of the forelimb digits and wrist (Whishaw et al., 1998; Morris et al., 2011, 2015).

In trained cats, movement disorders like ataxia, dysmetria, and difficulty in moving the distal regions of the forelimb appear after lesions to the RN (Sybirska and Górska, 1980). Similar results have been reported in rats trained to reach food pellets within a restricted space, which involves precise movement of the forelimb. In such experiments, complete lesions of the RN or lesions restricted to the parvocellular or magnocellular regions showed that the animals had mobility problems and could not perform precise movements, such as supination and pronation of the paw. They also exhibited deficiencies in digit movements (e.g., arpeggio) and had difficulty coordinating catching, aiming, and reaching efficiently (Whishaw et al., 1990, 1998; Morris et al., 2011, 2015).

The cerebral cortex controls RN activity by maintaining basal depolarization and modulating the ability to respond to other inputs received by RN neurons (Humphrey and Rietz, 1976; Tsukahara et al., 1983; Massion, 1988), as well as by facilitating motor-associated responses (Larsen and Yumiya, 1980). The cortico-rubral tract is excitatory in nature (Tsukahara et al., 1968), and its acting neurotransmitter is glutamate. This has been demonstrated by the presence of all the subunits that comprise the ionotropic receptors of glutamate, such as NMDA, AMPA, and Kainate receptors, and the diversity in RN cell responses (Bromberg et al., 1981; Minbay et al., 2017). Slow-conducting neurons of the pyramidal tract excite RN neurons, regulating their basal activity, while the neurons of the fast-conducting pyramidal tract make contact with the intrarubral interneurons, causing their inhibition (Tsukahara and Kosaka, 1968; Otero Costas and Lamas, 1982). These results suggest that the corticorubral tract is not only a direct effector of movement but also a regulator of the inputs of other structures that will affect the RS tract (Brown, 1974). In this way, the corticorubral tract participates in the initiation and termination of voluntary movements and regulates the basal activity of the RN, since it controls the excitability of incoming signals, especially those corresponding to the contralateral interposed nucleus of the cerebellum (Massion, 1988; Canedo, 1997).

In addition to the role of the RN in motor control, responses to sensory stimulation such as touch, proprioception, and pressure on the limbs have also been described. This is explained by the existence of a direct spinorubral pathway, which has been demonstrated in cats but has yet to be examined in rodents (Padel et al., 1986, 1988; Houk, 1991). Additionally, it has been described in rodents that stimulation of the RN induces analgesia (Prado et al., 1984). This analgesic response has been related to RN connections with the descending antinociceptive system, which includes the raphe nucleus and lateral reticular nucleus (Gwyn and Flumerfelt, 1974; Larsen and Yumiya, 1980; Basile et al., 2021). However, it remains to be determined if direct RS projections modulate nociceptive input at the segmental level.

## Corticospinal System

The CS system is undoubtedly better studied than the RS system. Using different anterograde and retrograde labeling techniques, Kuypers and Ugolini (1990) and Kuypers (2011) examined the neuroanatomy of the CS pathway and highlighted the relevance of its termination patterns by identifying its projection targets. The CS pathway originates mainly from motor and somatosensory areas of the brain and terminates in the spinal cord. Pioneering studies revealed that CS projections participate in basic sensorimotor functions such as the descending control of afferent inputs (Eguibar et al., 1994) and spinal reflexes (Evarts and Tanji, 1976; Wall and Lidierth, 1997), as well as excitation and inhibition of motor neurons (Jankowska et al., 1976; Alstermark, 1992; Porter and Lemon, 1993). More recently, the role of the CS system in sensory control has been described studying spino-cortico-spinal feed-forward sensitization loop that is crucial for controlling tactile sensation in normal conditions and allodynia in neuropathic pain states (Liu et al., 2018). This indicates that, in addition to the motor functions, the CS system has an important role modulating sensory information and thus an integral role in sensorimotor integration (Moreno-López et al., 2016).

The CS tract anatomy has some variations depending on the species. In rodents, CS axons from the cortex converge in the corpus callosum, course through the internal capsule, reach the pyramids, and decussate about 90% of axons (pyramidal decussation), continuing the path to the spinal cord contralateral to their origin cortex. The remaining 10% of axons do not decussate and continue the course ipsilaterally with respect to their origin cortex (Lemon and Griffiths, 2005; Lemon, 2008; Oudega and Perez, 2012). Axons descend to the spinal cord mostly *via* the dorsal funiculus and partly *via* the lateral and ventral funiculi (Joosten et al., 1992; Brösamle and Schwab, 1997). CS terminations densely innervate the dorsal horn and intermediate zone of the gray matter in the spinal cord, which is probably related to the descending control of sensory afferent inputs and limb muscle control (Lemon, 2008).

In primates, CS axons also course through the corona radiata and internal capsule, and between 75 and 95% decussate in the pyramids. The remaining 5–25% of axons do not decussate and continue their course. Axons descend to the spinal cord mostly through the lateral funiculus and minimally through the ventral funiculus (Lemon and Griffiths, 2005; Lemon, 2008; Oudega and Perez, 2012). Although CS terminations also innervate the dorsal horn and intermediate zone, many CS innervations reach the ventral horn and contact motoneuron pools related to the control of distal limb muscles (Lemon, 2008).

Despite a certain highly conserved organization of the CS tract through different species, there are important variations related to fine movement of the extremities and digits, since these types of movements emerged at different times throughout evolution (Iwaniuk and Whishaw, 2000; Lemon, 2008). One of the most notable differences is the presence or absence of monosynaptic cortico-motoneuronal (CM) projections between primate and non-primate species (Rathelot and Strick, 2006). For example, some studies show that there are no CM connections in species such as mice, rats, raccoons, and cats, even in primates such as marmosets or lemurs (Illert et al., 1976; Gugino et al., 1990; Yang and Lemon, 2003; Alstermark and Ogawa, 2004; Lemon and Griffiths, 2005). On the other hand, it has been reported that some primates, like the capuchin monkeys, macaques, and apes, exhibit a more developed CM system with more abundant projections (Bortoff and Strick, 1993; Lemon and Griffiths, 2005; Kuypers, 2011).

Collecting published data, Heffner and Masterton (1975) investigated the variations of the CS tract among 69 species of mammals and their respective degree of digital dexterity, an arbitrary measure that evaluates the use of digits in grasping movements (Napier, 1956, 1961). The authors used parameters such as the number of fibers per tract, the penetration down the spinal cord, and the lamina of axon terminals together with the dexterity index. For example, non-primate species such as Carnivora (cats, dogs, and raccoons), Chiroptera (bats), and Rodentia (rodents) have a dexterity of 1–3, whereas the primate species (monkeys, apes, and humans) have a dexterity level of between 4 and 7. Thus, one idea proposes that the difference in digital dexterity between species is due to the presence and density of CM projections, which allow a direct control of the cerebral cortex over the motor neurons of the spinal cord (Heffner and Masterton, 1975; Porter and Lemon, 1993; Rathelot and Strick, 2006; Lemon, 2008; Yoshida and Isa, 2018). This CM system is absent in non-primate mammals; however, it becomes more prominent in primate species. In fact, these CM projections are considered to be exclusive to primates, but not all primates have them (Lemon and Griffiths, 2005; Lemon, 2008; Yoshida and Isa, 2018).

The functional observation in dexterity evolution correlates well with the anatomical distribution of the CS tract axon terminations in all gray matter of the spinal cord (Armand, 1982). In rodents and marsupials, CS tract endings are largely distributed in the dorsal horn. However, ascending the phylogenetic scale, in carnivores and primates, the CS terminations shift progressively invading the intermediate zone and ventral horn, ultimately forming increasing numbers of synaptic terminations directly on the motoneurons themselves in higher primates (for a review, see Schieber, 2007).

## Motor Deficits and Plasticity After Corticospinal and Rubrospinal Lesions

Upon injury to the CS tract, outgoing motor and incoming sensory projections can be compromised, leading to partial or complete motor deficits depending on the location (cortex, internal capsule, brainstem, or spinal cord) and size of the lesion (Oudega and Perez, 2012; Serradj et al., 2017; Isa et al., 2019). For example, Weidner et al. (2001) reported permanent impairment in the performance of a pellet retrieval task in rats with complete transections of the CS tract at the level of the pyramids. Furthermore, spontaneous sprouting of the ventral ipsilateral CS tract axons occurs after transection of the dorsal CS tract. Similarly, lesions of the CS tract at the level of the pyramids in monkeys produced permanent damage of the precision grasping (Lawrence and Kuypers, 1968). However, after some lesions at the cortical (premotor areas and sensorimotor cortex) or spinal level, it is possible to observe relatively good recovery (Morecraft et al., 2015; Darling et al., 2018; Isa et al., 2019) that can be attributed to the reorganization and plasticity of cortical and spinal circuits (Oudega and Perez, 2012; Serradj et al., 2017; Mohammed and Hollis, 2018; Isa et al., 2019).

It is well documented that, although recovery is incomplete, when there is a lesion in the RN, there is compensation for the CS tract. Conversely, when there is a damage to the CS tract, there is compensation on the cortico-rubro-spinal part (Ishida et al., 2019). This has been seen for some types of movement, such as skillful movements, but not for others, such as locomotion (Kennedy and Humphrey, 1987; Kennedy, 1990; Kanagal and Muir, 2009; Bertucco and Dayanidhi, 2014). The RS and CS tracts are closely related and converge at segmental level modulating interneurons and propriospinal neurons (Illert et al., 1976, 1977; Alstermark et al., 1981; Canedo, 1997). In cats, a spinal cord disynaptic circuit of cortico-motoneuronal and rubro-motoneuronal excitation has been described in the C3–C4 segments which activates motoneurons controlling forelimb muscles. This is a pathway of rapid motoneuronal activation and a point of convergence for the descending pathways (RS, CS, reticulospinal, and tectospinal) that preferably modulates faster rather than slower motoneurons (Illert et al., 1976, 1977; Alstermark et al., 1981). On the other hand, propriospinal interneurons activated by cutaneous and muscle afferents are monosynaptically excited by descending CS and RS pathways, suggesting that the descending commands for the execution of a movement can be modified by sensory information and indicating that motor commands can be affected by changes in the internal and external environments, modifying the original command of the descending pathways (Illert et al., 1976, 1977).

Despite some differences in movement types, the CS and RS tracts contribute jointly for skillful forelimb movements, including reaching and manipulating food as well as locomotion (Whishaw et al., 1993; Kanagal and Muir, 2009; Morris and Whishaw, 2016). Moreover, selective injuries of CS and RS tracts experimentally produce movement deficits in trained animals. In this way, arpeggio, or pronation of the hand, seems to be more related to the RS tract, whereas reach and grip are associated with the CS tract (Whishaw et al., 1998; Morris et al., 2011). However, even after removing the CS or RS tract, wide repertoires of movements remain (Illert et al., 1977), indicating that various subcortical nuclei actively participate in motor control. This also suggests, with an evolutionary constraint, that the function of the cortex has evolved regulating circuits of pre-existing motor systems and pathways and not as an independent movement execution system. Therefore, motor control must be understood as a set of descending systems that converge modulating primitive segmental neuronal circuits.

Studies in humans have shown that the RN is involved in the improvement of mobility in patients with CS tract damage. In this way, Yeo and Jang (2010) found an increased factor of anisotropy, suggesting major neuronal activity in the RN after pyramidal tract injury. Similarly, patients who suffer a stroke in cortical motor areas exhibit increased compensatory activity in the bilateral RN of the injured hemisphere which correlates with the degree of mobility recovery (Rüber et al., 2012; Takenobu et al., 2013). Further, an increase in the anisotropy factor of the cortico-rubro-spinal tract in the unaffected hemisphere of patients with severe CS damage has been found (Jang and Kwon, 2015), and patients with chronic stroke showed a higher anisotropy factor in the unaffected RN in severe and complete CS tract injury (Kim and Lee, 2018). On the other hand, the RN seems to be a compensatory mechanism of movement control in Parkinson’s disease (Philippens et al., 2019). All these results suggest a compensatory role of RS connections in the motor recovery of patients with a CS tract injury.

## Segmental Neuronal Circuits Modulated by CS and RS Systems

Experimental evidence confirms that both CS and RS systems drive distinct segmental neural circuits that are part of the sensory and pre-motor pathways. Better is known about the identity of the spinal cord interneurons that are under cortical control, and it is now accepted that the CS system is functionally and anatomically segregated (Moreno-López et al., 2016; Olivares-Moreno et al., 2017; Steward et al., 2020). The target interneurons of the CS tract include different classes of pre-motor interneurons (Alstermark and Ogawa, 2004; Ni et al., 2014; Ueno et al., 2018), interneurons mediating primary afferent depolarization of cutaneous and Ib (tendon organs) but not Ia (muscle spindle) afferents (Rudomin and Schmidt, 1999) and interneurons regulating ascending proprioceptive information (Hantman and Jessell, 2010). Recent findings reveal that CS neurons in the sensorimotor cortices differentially control skilled movements through CS interneuron circuits (Ueno et al., 2018).

Unfortunately, the study of the neuronal segmental circuits that are under control of RS projection has been neglected in recent years. However, classic studies measuring the intraspinal threshold and intracellular recordings of single afferent fibers (Rudomín et al., 1983; Rudomin et al., 1986; Jiménez et al., 1987) confirmed the initial observations of Eccles and Lundberg (Eccles et al., 1962; Lundberg, 1964) describing that, similar to the pyramidal tract, RS projections produce no primary afferent depolarization of Ia afferents, but was instead inhibit the primary afferent depolarization produced by other afferents; nevertheless, Ib afferents are depolarized by both CS and RS tracts. Moreover, both descending systems are able to reduce the tonic presynaptic inhibition of muscle spindle afferents during voluntary movement (Iles, 1996; Rudomin and Schmidt, 1999). In addition to the modulation of sensory information flux, RS projections can influence pre-motor interneurons (Kostyuk and Skibo, 1975), as well as some motoneuronal groups (Küchler et al., 2002) similar to the CS system. However, it is necessary to further analyze the precise segmental interneurons that are directly driven by the RN.

On the other hand, some differences in the population activity between CM and rubromotoneuronal (RM) influences, on single motor units in monkeys, have been reported (Fetz et al., 1989). RM cells facilitate more muscles per cell and with a shorter latency than CM cells. In general, CM cells facilitate both extensor (48%) and flexor (51%) muscles. In contrast, RM cells facilitate more extensor muscles (78%) than flexor (22%) muscles (Fetz et al., 1989; Cheney et al., 1991). On the other hand, classical experiments in cats have described that stimulation of the RN produces both flexion and extension in forelimbs as well as hindlimbs (for review, see Massion, 1967). Particularly interesting is the fact that the stimulation of the RN commonly produces EPSPs and IPSPs on both, flexor and extensor motor neurons, but with a clear predominance of the excitatory action on the flexor muscles (Hongo et al., 1969). The biased contribution of RS tract on flexor control contrasts with observations in which “anatomical extensors” of forelimb muscles (e.g., extensor digitorum communis) are activated by RN stimulation (Rho et al., 1999). However, it is necessary to point out that wrist extensors are “physiological flexors,” indicating that RS facilitation of upper limb physiological flexors in cats, and anatomical extensor in primates, could be considered analogous and only depends on the way in which different muscles are classified as flexors or extensors.

A differential cortical control over the flexor and extensor muscles has been also proposed. For example, in forearm muscles, Cheney and Fetz (1980) found greater CM input to extensors rather than flexors. In elbow muscles, Palmer and Ashby (1992) reported greater cortical input to flexors than to extensors. Moreover, van Kan and McCurdy (2001) described strong correlation between duration, amplitude, and velocity of metacarpi-phalangeal extension with magnocellular RN neuronal discharges. On the other hand, classical experiments in cats have described that stimulation of the RN produces both flexion and extension in forelimbs as well as in hindlimbs (for review, see Massion, 1967). The previous evidence suggests that it is not possible to affirm that RS projections could compensate a loss of CS function after CNS damage.

The differences between CS and RS influences become clearer under conditions of pathology or injury. For example, in patients with amyotrophic lateral sclerosis, a condition that affects the cortex and corticofugal fibers, muscles controlled by α-motoneurons and CM cells are differentially affected. Ludolph et al. (2020) compared strength between elbow extensors (triceps) and flexors (biceps), reporting greater relative weakness in flexors relative to elbow extensors; in addition, they also reported greater relative weakness of extensors vs hand flexors. Similarly, in participants with cervical spinal cord damage (tetraplegia) who received transcranial magnetic stimulation, motor-evoked potentials were smaller in triceps relative to controls, suggesting that there is less CS input to the elbow extensors (Sangari and Perez, 2020). Furthermore, in monkeys with CS tract lesions, Zaaimi et al. (2012) reported increased facilitation of motor neurons related to elbow flexors but not in those with connections to extensors. This supports the findings of Belhaj-Saïf and Cheney (2000), who reported increased facilitation of flexors and reduced facilitation of both wrist and digit extensors following incomplete CST injury. Those authors point to a rebalancing in descending motor information from brainstem projections toward flexor rather than extensor muscles after CS tract lesions (Belhaj-Saïf and Cheney, 2000; Zaaimi et al., 2012).

These studies support the idea that after CS tract damage, the muscles with the greatest CM influence show greater weakness, and there may be RM involvement in compensatory (rebalancing) mechanisms for motor alterations (Palmer and Ashby, 1992; Belhaj-Saïf and Cheney, 2000; Zaaimi et al., 2012; Ludolph et al., 2020; Sangari and Perez, 2020). In this way, more recently it has been described in Celsr3| Emx1 mutant mice model, where CST is specifically and fully absent, that axonal projections from RN to the spinal cord are increased and lesions of the RS tract lead to defective forelimb use, with almost no recovery. In contrast, the lesion of the RS tract in normal mice results in partial motor deficits that recover rapidly. Moreover, there are no changes of spinal projections from vestibular nuclei or the reticular formation, meaning that the main plasticity in Celsr3| Emx1 model is due to the RS tract (Han et al., 2015). These results indicate that, in the mutant, the RS tract palliates defective CS tract function in movement control.

## Concluding Remarks

Both old RS and new CS descending systems regulate diverse segmental neuronal circuits involved in sensorimotor integration. Before the emergence of the cerebral cortex in ancient vertebrates, the RS system acquired a fundamental role for movement and motor control. However, in mammals, the cerebral cortex takes a superior hierarchical role controlling pre-existing subcortical structures including the RN, generating specializations like the CM systems of superior primates. The RN has been considered a vestigial structure in humans; however, the compensatory activity of a phylogenetically ancient structure like RN, observed after lesions in the new CS system, indicates that is not the case (Jones and Adkins, 2015). Thus, it is tempting to propose that the cerebral cortex masks some of the original functions of the RN, which only became evident after lesions of the pyramidal system.

The experimental study of the RS system has been abandoned for a long time. Nevertheless, it is necessary to shift our focus toward an integrative study of motor systems—particularly the RN since it could potentially subserve motor recovery associated with CNS damage.

## Author Contributions

RO-M and GR-P edited the manuscript. All authors drafted the manuscript and approved the final version.

## Conflict of Interest

The authors declare that the research was conducted in the absence of any commercial or financial relationships that could be construed as a potential conflict of interest.
